# Robust Statistical Approaches for RSS-Based Floor Detection in Indoor Localization

**DOI:** 10.3390/s16060793

**Published:** 2016-05-31

**Authors:** Alireza Razavi, Mikko Valkama, Elena Simona Lohan

**Affiliations:** Department of Electronics and Communications Engineering, Tampere University of Technology, Tampere 33720, Finland; mikko.e.valkama@tut.fi (M.V.); elena-simona.lohan@tut.fi (E.S.L.)

**Keywords:** indoor localization, floor detection, RSS-based localization, robust regression, weighted centroid localization, trilateration

## Abstract

Floor detection for indoor 3D localization of mobile devices is currently an important challenge in the wireless world. Many approaches currently exist, but usually the robustness of such approaches is not addressed or investigated. The goal of this paper is to show how to robustify the floor estimation when probabilistic approaches with a low number of parameters are employed. Indeed, such an approach would allow a building-independent estimation and a lower computing power at the mobile side. Four robustified algorithms are to be presented: a robust weighted centroid localization method, a robust linear trilateration method, a robust nonlinear trilateration method, and a robust deconvolution method. The proposed approaches use the received signal strengths (RSS) measured by the Mobile Station (MS) from various heard WiFi access points (APs) and provide an estimate of the vertical position of the MS, which can be used for floor detection. We will show that robustification can indeed increase the performance of the RSS-based floor detection algorithms.

## 1. Introduction

Indoor localization is becoming more and more important in today’s wireless world. Being able to achieve accurate ubiquitous localization on hand-held battery operating mobile devices in both indoor and outdoor environments would open the window to many new Location Based Services (LBS). Despite the fact that outdoor global localization solutions exist nowadays with the help of Global Navigation Satellite Systems (GNSS), global solutions for indoor localization are still hard to find. Fingerprinting (FP) approaches can solve the indoor localization problem locally [1], but such solutions are expensive and rather computationally expensive to be used on a global scale, meaning over several countries or the entire world. In the fingerprinting-based methods, the location service providers construct a fingerprint database, transfer this database to the mobile station (MS), and the MS then computes its location based on similar fingerprints. The fingerprint databases are typically very large since they do contain received signal strengths (RSSs) coming from various access points (APs) and in many points or coordinates within a building. Thus, if a global localization solution would use a fingerprinting approach, the fingerprint database transferred from the server to the MS would include the fingerprints from all essential buildings in the town (or the location area) where the mobile is situated. For example, assuming that we hear an average of 30 APs in each location point inside a building, that we take measurements from an average of 600 location points per building, that there are 25 important buildings (malls, shopping centers, hospitals, airports, *etc.*) in the location area where the mobile was identified by the network, then a total of 495,000 parameters would need to be stored in the database pertaining to that town and transferred to the mobile. The parameters are the fingerprints—namely, the (x,y,z) coordinates and the measured RSS values per coordinate. In addition, if these parameters are saved with a 32-bit accuracy, the database size of such a server provider for the particular town of our example would be around 15.86 Mbits. Assuming that many users are simultaneously downloading this amount of positioning data from the network, the network would soon become very crowded and the data capacity for wireless communications would be tremendously decreased in order to support the positioning requests. This could easily create a bottleneck on the server side; thus, it is very important to find statistical methods to use instead of fingerprinting in order to decrease the amount of parameters to be sent to the mobile, and thus the data usage for positioning purposes.

There are two alternative ways to address these problems: one is to compute the user’s location entirely at the server side, and the second is to use a probabilistic approach, where what we transfer to the mobile is a smaller number of parameters, extracted in a statistical way from the database. The first solution, which is entirely network-based, suffers from several drawbacks: it offers the user location with some delays (due to the propagation delays between the MS and the network server), and with reduced accuracy (due to the fact that the MS might move between the moment when it sent its location request to the server and until the location computed by the server reaches it). Additionally, since the user location needs to be provided on a continuous basis and for users from different towns, if a significant number of users send their location requests to the location server, then the amount of processing required at the server side also becomes quickly unfeasible.

The second approach, which alleviates all these problems (the need for transferring huge databases with fingerprints and the problem of dealing with a huge amount of data at the server side when the positioning is purely network-based), is the approach addressed in this paper. Here, the positioning and floor estimation is done entirely by the mobile device, and the location server transfers only a small amount of data to the MS. In the probabilistic approaches, instead of transferring the fingerprints database, we only transfer a few parameters per AP in a building (five parameters per AP, as in path-loss model Equation (4)). Taking the previous example and assuming an average of 150 APs per building, the server would need to transfer to the MS only 18,750 parameters, or 0.6 Mbits, which would take at most 12 s with the average speed of 50 kpbs. This time already becomes comparable with the cold start-up times in modern GPS receivers. Therefore, the mobile-computing localization algorithms developed based on this approach are highly suitable for the mobile devices of tomorrow which support localization and navigation features. The algorithms developed in this paper lie within this approach.

Due to the unavailability of true channel model, as well as the distribution of noise/modelling errors, most of the probabilistic approaches—whether explicitly or implicitly—employ the standard least-squares (LS) method, which is optimal in the maximum-likelihood sense under the Gaussianity assumption. This assumption may impose significant errors, especially when the noise/modeling errors expose outliers or have tails heavier then Gaussian distribution.

In this paper, we employ M-estimators as a means for robustifying the probabilistic positioning methods, when the model of noise/modelling errors deviates from Gaussianity. The term *M*-estimation is used because this class of estimators can be regarded as a generalization of *M*aximum-likelihood estimation [2]. We show how M-estimates can be deployed in four existing positioning methods, namely Weighted Centroid Localization (WCL) [3], linear and nonlinear trilateration [4], and deconvolution- based method [5]. The comparison between the performance of the ordinary and the robustified version for each method is studied using some numerical results, which verify that the M-estimates can provide a higher degree of robustness for these methods.

The potential applications are when a user wants to use his/her smart phone to localize himself/herself inside a multi-floor building (e.g., a shopping mall) that the user may visit for the first time, and possibly guide him/her to a certain favorite place (e.g., the closest shoe store) inside the building. The application that the user uses on the smart phone can have a complete map of the shopping mall, including the 3-dimensional coordinates of all the stores and places inside the building, but to be able to guide him/her to their favorite place it must be able to first localize itself. We would mention here that the focus of this work is on the *z*-axis localization, rather than the three-dimensional (x,y,z) localization. Recently, there has been an increasing interest in estimating the *z* coordinates due to the fact that finding the correct floor is more important than (x,y) coordinates, as few meters error in estimation of *z*-coordinates may lead the mobile user to the wrong floor in positioning [6,7,8].

The novelty of our paper is two-fold: first, we present four innovative robustification methods for mobile-based floor detection in wireless positioning, and second, we validate our algorithms through measured and simulated data and show their feasibility in realistic multi-floor scenarios. We point out that the goal of this paper is not to compare different RSS-based localization algorithms (or their robustified counterparts) but rather to show that robustification can improve the performance of each method.

The organization of this paper is as follows: in Section 2 we describe the system model and briefly explain two celebrated methods of indoor localization—namely, the fingerprinting approach and the path-loss approach. The theoretical contribution of the paper is introduced in Section 3, where after describing the *M*-estimates of regression, we will exploit it to robustify four existing indoor localization approaches. Section 4 is devoted to the performance analysis of the proposed robust localization approaches, based on numerical real-life measurements, and a simulation experiment is provided in Section 5. Finally, we conclude the paper in Section 6.

**Notations:** Throughout this paper, matrices are denoted by capital boldface letters and vectors and tuples are denoted by small boldface letters. = denotes the equality and ≜ denotes the definition. Besides, Table 1 summarizes the most important notations used for denoting quantities used in positioning in this paper.

## 2. System Model and Background on Indoor Localization Methods

Consider a localization system equipped with $N_{AP}$ positioning signals (e.g., RSS values received from APs). During the offline phase, the positioning signals are collected in $N_{AP}\times 1$ *measurements vectors* $m_{i}≜{\left[P_{i,1},P_{i,2},\ldots ,P_{i,N_{AP}}\right]}^{T},\;i=1,\ldots ,N_{FP}$, where $N_{FP}$ is the number of fingerprints collected in the building and $P_{n,ap}$ is the RSS received from access point ap at *n*-th collected fingerprint. The corresponding known 3-D location of $m_{i}$ is denoted by ${\underline{c}}_{i}≜\left({\underline{x}}_{i},{\underline{y}}_{i},{\underline{z}}_{i}\right),\;i=1,\ldots ,N_{FP}$. We will use the stored data, $D≜\{m_{i},{\underline{c}}_{i},\;i=1,\ldots ,N_{FP}\}$, for indoor localization. In the following, we briefly explain two of the famous methods—a nonparametric method and a parametric method—for indoor localization based on the stored data. Then, in Section 3 we will introduce some robust methods for indoor localization.

### 2.1. Fingerprinting Localization

In the fingerprinting approach [9,10,11], the fingerprints $\left\{m_{i},{\underline{c}}_{i},\;i=1,\ldots ,N_{FP}\right\}$, are stored and directly used for localization purposes.

Assume that a Mobile Station (MS), located at unknown coordinates c=(x,y,z), observes a positioning vector $m_{\mathrm{MS}}≜{\left[p_{1},p_{2},\ldots ,p_{N_{AP}}\right]}^{T}$, where $p_{ap},\;ap=1,\ldots ,N_{AP}$ is the RSS received from the ap-th AP during the online phase. The basic 1-Nearest Neighbor (1-NN) fingerprinting (FP) approach estimates the location of the MS as

(1) $${\hat{c}}_{\mathrm{FP}}={\underline{c}}_{j}$$

where

(2) $$j=\underset{i\in \{1,\ldots ,N_{FP}\}}{\arg}\min d\left(m_{\mathrm{MS}},m_{i}\right)$$

and d(·,·) is a dissimilarity measure which is determined based on our assumption for noise. For instance, if we assume that the noise which deviates the $m_{\mathrm{MS}}$ from $m_{i}$ is i.i.d white Gaussian, then $d(m_{\mathrm{MS}},m_{i})$ is simply the squared Euclidean distance between $m_{\mathrm{MS}}$ and $m_{i}$; *i.e.*,

(3) $$d\left(m_{\mathrm{MS}},m_{i}\right)={∥m_{\mathrm{MS}}-m_{i}∥}_{2}^{2}$$

In general, the fingerprint-based localization approach is a *pattern matching* approach [9,10,11] rooted in pattern recognition [12], which tries to match the pattern $m_{\mathrm{MS}}$ observed by MS to the examples ${\left\{m_{i}\right\}}_{i=1}^{N_{FP}}$ collected in the training data set, and chooses the location of the less-dissimilar example (fingerprint) as the location of the MS. In this regard, each element of the measurements vector $m_{i}$ is a *feature* of the location ${\underline{c}}_{i}$. On the other hand, any measured signal which depends *only on the measurement location* (regardless of noise, shadowing, and other uncertainties) can be regarded as a *feature* of that location and used for localization using the fingerprinting scheme.

The main problem with the fingerprinting approach is the huge amount of data which must be stored by servers and transmitted to the MS to localize itself when $N_{FP}$ is a large number. The situation becomes even more severe when the fingerprints are being collected all the time. If we want to use fingerprinting methods for localizing the mobile device, it can only be done on the server side. Due to the limited processing capability and power supply on most mobile devices, they are not capable of storing and processing that huge amount of data [13]. Furthermore, transmitting such an amount of fingerprinting data from the server to the mobile device takes a lot of time, which makes localization by mobile devices impractical.

### 2.2. Path-Loss Approach for Indoor Localization

To cope with this problem, a well-known alternative is to use the so-called parametric approaches for indoor localization. In parametric approaches, we use a parametric model for finding the MS location. The parameters of the model are then estimated based on the training data (fingerprinting data) and the MS only stores these parameters and uses them for localization by applying them to the online received positioning vector. In the following, we briefly describe a parametric approach for indoor localization based on the path-loss model.

Any RSS-based localization and floor detection method includes two stages: a training stage and an estimation stage. In the training stage—done either in a dedicated/manual mode or in a crowdsource mode—the data is collected building by building, and the following observations are stored on a server database: $D=\{\left({\underline{x}}_{i},{\underline{y}}_{i},{\underline{z}}_{i}\right),P_{i,ap},i=1,\cdots ,N_{FP},ap=1,\cdots ,N_{AP}\}$, where $\left({\underline{x}}_{i},{\underline{y}}_{i},{\underline{z}}_{i}\right),i=1,\cdots ,N_{FP}$ are the 3D coordinates where the measurements were taken within a certain building, $N_{FP}$ is the total number of fingerprints measured in a building, $N_{AP}$ is the total number of AP per building, and $P_{i,ap},i=1,\cdots ,N_{FP},ap=1,\cdots ,N_{AP}$ is the measured RSS for the *i*-th fingerprint, coming from the ap-th access point.

In the path-loss approach for the localization (one of the main parametric approaches for localization), the target is to extract a sub-set S of relevant information starting from the available database D. The steps are as follows:

(1) Estimate the unknown AP locations (if there is a known AP location, this can replace the estimated ones). The estimation can be done either via averaging over the positions of a few of the strongest fingerprints (*i.e.*, where that AP was heard with strongest power) or via the use of a weighted approach, such as the one presented in [14], or by applying a two-step deconvolution process, as described in [5]. Our studies showed that the weighted approach gives slightly better results than the other two; thus, it will be selected in our analysis. Since AP location is done in 3D plane, after this step, there will be three parameters to be stored per AP regarding AP position.

(2) Associate an underlying path-loss model with the measured RSS and estimate the model parameters via certain statistical methods. In our paper, we will discuss two path-loss models: the classical slope-based path-loss model [5] with two additional parameters per AP (transmit power and slope coefficients), and a new simplified path-loss model which focuses on the *z*-dimension only and has only one parameter. Thus, the number of extracted parameters per AP is five or six, according to the underlying path-loss model.

The *traditional path-loss model* is based on free space wave propagation [15], and involves two modeling parameters per AP: $\Theta_{AP}=\left[P_{T_{ap}}\;n_{ap}\right]$, where $P_{T_{ap}}$ is the ap-th AP transmit power and $n_{ap}$ is the path-loss coefficient of the ap-th AP. Those two parameters are related to the RSS via:

(4) $$P_{i,ap}=P_{T_{ap}}-10n_{ap}log_{10}d_{i,ap}+\eta_{i,ap}$$

where $P_{T_{ap}}$ is the ap-th AP transmit power and $n_{ap}$ is the path-loss coefficient of the ap-th AP, and $\eta_{i,ap}$ is a noise factor, typically assumed Gaussian distributed, of zero mean and standard deviation *σ*. The noise is typically due to shadowing, fading, and measurement errors: $\eta_{i,ap}\propto N\left(0,\sigma^{2}\right)$. Above, $d_{i,ap}=\sqrt{{\left({\underline{x}}_{i}-x_{ap}\right)}^{2}+{\left({\underline{y}}_{i}-y_{ap}\right)}^{2}+{\left({\underline{z}}_{i}-z_{ap}\right)}^{2}}$ is the Euclidean distance between the ap-th AP and the *i*-th measurement point. The above model can be re-written in matrix form as:

(5) $$P_{\mathrm{ap}}=H_{\mathrm{ap}}\Theta_{\mathrm{ap}}^{⊤}+n$$

where $P_{ap}≜{\left[P_{1,ap}\;P_{2,ap}\;\cdots \;P_{N_{FP},ap}\right]}^{⊤}$ is the vector with power fingerprints in logarithmic scale coming from the ap-th access point, ${}^{⊤}$ is the transpose operator, n is a Gaussian distributed $N_{FP}\times 1$ vector with elements $\eta_{i,ap}$ and

(6) $$\begin{array}{c} H_{ap}≜\left[\begin{array}{cc} 1 & -10log_{10}d_{1,ap}\\ & \cdots \\ 1 & -10log_{10}d_{N_{FF},ap} \end{array}\right] \end{array}$$

### 2.3. Memory Complexity of Model-Based Approaches Versus Fingerprinting Approach

We live in the era of Big Data and the need for algorithms which can cope with the huge volume of data and extract insight from it has been a major challenge in almost all data science areas, including wireless localization, for the past few years and will remain a challenge for a foreseeable future. While distributed and cloud-based algorithms remain a clear candidate in many cases, there are scenarios, like what we have here for indoor localization, that the technical limitations (here, the fact that a mobile device should perform the localization task in real-time and cheaply, and therefore cannot get help from other nearby devices or use cloud services).

To address the memory complexity issue we provide a brief comparison between the complexity of fingerprinting approach and model-based approaches in terms of the memory size required for performing localization in the mobile side. In fingerprinting approach the size of data we need to store in the mobile device is proportional to $N_{fp}\times N_{ap}$ as we need to store all fingerprinting vectors which are of size $N_{ap}$. However in model-based methods (such as the ones proposed in this paper and also in their original non-robust counterparts) the size of data that we need to keep is just proportional to the number of APs, *i.e.* $N_{ap}$. For example in the above-mentioned path-loss approach we need to store $5N_{ap}$ parameters, *i.e.*, the 3 coordinates of each AP plus their transmit power and their associated path-loss exponents. In Weighted Centroid Localization [3,14,16,17] approach, as well as its robust version which will be introduced in the next section, we only need to store the 3 coordinates of each AP which means a total of $3N_{ap}$ parameters. This is a significant reduction in the size of data as usually $N_{fp}$ can be a huge number but $N_{ap}$ is limited by the hardware cost employed in the building for internet coverage which is typically tens to hundreds maximum.

We remark that all the robust approaches introduced in this paper have the same memory complexity as of their non-robust counterparts and do not increase the size of the data needed to store in the mobile side.

## 3. Robust Floor Estimation Algorithms

In this section, we introduce four robust floor estimation algorithms. We first review the *M*-estimates of regression—a method for robust estimation of parameters in a linear regression problem—and then, based on that, robustify four existing indoor localization algorithms for floor estimation.

Consider the linear regression problem

(7) y=Hx+ε

where H is the matrix of regressors (for example matrix $H_{ap}$ in Equation (5)), y is the n×1 noisy observation vector, x denotes the k×1 parameter vector for which we are going to find an estimate, and ε denotes the error vector whose entries are assumed to be i.i.d. from a symmetric continuous distribution with an unknown scale parameter *s*.

**Figure 1:**
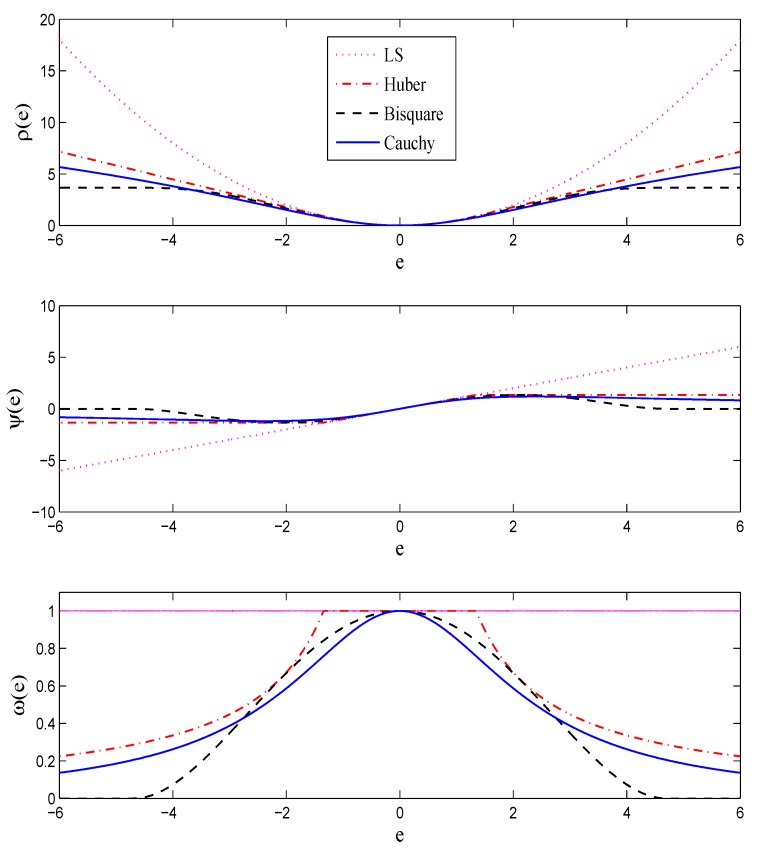
The functions ρ(e) (upper plot), ψ(e) (middle plot) and w(e) (lower plot) for the ordinary least-squares and three commonly-used robust functions, namely Huber, Bisquare and Cauchy.

Let $e_{i}=e_{i}\left(x\right)=y_{i}-h_{i}^{⊤}x$ denote the *i*-th residual for a candidate vector x, where $y_{i}$ is the *i*-th entry of y, and $h_{i}$ is the *i*-th row of matrix H. At this point, assume that the scale parameters are known. The ordinary least-squares (LS) tries to minimize $\sum_{i}e_{i}^{2}$, which yields an unstable solution in the presence of outliers or heavy-tailed noise. The *M*-estimators reduce the effect of outliers and heavy-tailed noise by replacing the sum of squared residuals by the following objective function

(8) $$\sum_{i=1}^{n}\rho \left(e_{i}\left(x\right)/s\right)$$

where ρ(·) is a symmetric, convex, positive-definite function; see, e.g., the upper plot in Figure 1.

Let $\psi =\rho^{\prime }$ be the derivative of *ρ*. Then, the minimizer of Equation (8) is the solution to the following equation

(9) $$\sum_{i=1}^{n}\psi \left(e_{i}\left(x\right)/s\right)h_{i}^{⊤}=0$$

Defining $w\left(e_{i}\right)≜\psi \left(e_{i}\right)/e_{i}$ and denoting $w_{i}≜w\left(e_{i}/s\right)$, Equation (9) can be re-written as

(10) $$\sum_{i=1}^{n}e_{i}\left(x\right)w_{i}h_{i}^{⊤}=0$$

Since $w_{i}$ is iteself a function of $e_{i}$, to solve Equation (10) we use an iterative algorithm called *iterative re-weighted least squares (IRLS)* [18,19], where in each iteration *t* an estimate ${\hat{x}}_{\left\{t\right\}}$ is computed from Equation (10) by assuming that $w_{i}$ is constant, then the residuals and weights are updated based on ${\hat{x}}_{\left\{t\right\}}$, which will be used in the next iteration for solving Equation (10) and finding the new estimate ${\hat{x}}_{\left\{t+1\right\}}$. This iteration continues until a stopping criterion is satisfied. Since the scale *s* is unknown in practice, it is commonly replaced at each iteration by a robust estimate $\hat{s}$ calculated from the current residuals. A commonly-used estimator for scale is the median absolute deviation (MAD) $\hat{s}=\mathrm{MAD}\left(e\right)=1.4286\cdot {\mathrm{median}}_{i}\left(\right|e_{i}-{\mathrm{median}}_{i}\left(e_{i}\right)\left|\right)$, which is the default choice in the robustfit routine of Matlab. From now on, we denote this iterative robust solution to the regression problem of Equation (7) as

(11) robustfit(H,y)

Choosing the weight function w(·), or equivalently ψ(·) and ρ(·), plays an important role here. In fact, the function *ρ* can be interpreted as $-\log f_{e}\left(e/s\right)$, which means that if the error term has distribution $f_{e}$, then the IRLS estimate coincides with the maximum likelihood (ML) estimate. In practice, we do not know the error distribution and we choose the weight function so as to alleviate the effect of large errors stemming from impulsive or heavy-tailed noise. This is accomplished by choosing bounded ψ(·) functions. Function ψ(·), sometimes called the *influence function* [20], determines the influence of a datum on the estimate. For example, in the LS case when ψ(e)=e, this influence is linear, which results in the non-robustness of the LS solution. On the other hand, by choosing a bounded *ψ*, the influence of large errors stemming from outliers or heavy-tailed noise is bounded, which results in the robustness of the estimate. Some commonly-used robust functions which will be used later in this paper for robustification of localization approaches are presented in Table 2 and Figure 1. As can be seen from Figure 1, all three robust influence functions are bounded. The constant *k* is called the *tuning constant*, which trades between the robustness and the efficiency when the noise is Gaussian; smaller values of *k* provide more robustness but are less efficient when the noise is normally distributed [2].

In followings, we show concrete examples of how these robustification functions can be applied to solve the 3D indoor localization problem.

### 3.1. Robust Weighted Centroid Localization

The weighted centroid localization (WCL) approach, first proposed for position estimation in wireless sensor networks [3], is a simple and low-complexity but promising localization approach. The position of the MS in the WCL approach is computed as the weighted average of the positions of APs heard by the MS. Denoting the set of all hearable APs by H and the (known) coordinates of APs by $c_{ap}≜\left(x_{ap},y_{ap},z_{ap}\right),\;ap=1,\ldots ,\left|H\right|$, the WCL-based estimate of mobile station coordinates is computed as

(12) $${\hat{c}}_{\mathrm{WCL}}=\frac{\sum_{ap\in H}w_{ap}c_{ap}}{\sum_{ap\in H}w_{ap}}$$

where $w_{ap}$ are weight functions. To weight shorter distances (nearer APs) more than higher distances, $w_{ap}$ may be chosen as [3]

(13) $$w_{ap}=1/{\left(d_{ap}\right)}^{g}$$

where $d_{ap}$ is the distance between the ap-th AP and the MS, and degree *g* is to ensure that remote APs still impact the position estimation [3].

Since $d_{ap}$ are not readily available, and also since RSS heard from AP ap is inversely proportional to $d_{ap}$, the weights $w_{ap}$ in Equation (12) can be replaced by RSS to obtain the following RSS-based formula for WCL [14,16,17]

(14) $${\hat{c}}_{\mathrm{WCL}}=\frac{\sum_{ap\in H}p_{ap}c_{ap}}{\sum_{ap\in H}p_{ap}}$$

where $p_{ap}$ is the measured RSS of AP number ap.

Equation (14) can be written independently for each coordinate. For instance, for the height coordinate (which is the coordinate that matters in the floor detection task), we have

(15) $${\hat{z}}_{\mathrm{WCL}}=\frac{\sum_{ap\in H}p_{ap}z_{ap}}{\sum_{ap\in H}p_{ap}}$$

To robustify the WCL approach, we first remark that Equation (15) can be written as

(16) $${\hat{z}}_{\mathrm{WCL}}=\arg\min_{z}\sum_{ap\in H}{\left|e_{ap}\left(z\right)\right|}^{2}$$

where $e_{ap}\left(z\right)≜\sqrt{p_{ap}}\left(z-z_{ap}\right)$.

The robust WCL is then obtained by replacing $|e_{ap}{\left(z\right)|}^{2}$ in the right-hand side of Equation (16) by the general cost function $\rho (e_{ap}\left(z\right)/s)$, which yields

(17) $${\hat{z}}_{\mathrm{RWCL}}=\arg\min_{z}\sum_{ap\in H}\rho \left(\frac{e_{ap}\left(z\right)}{s}\right)$$

where *s* is the scale parameter, which can be estimated as discussed in the previous section.

#### Implications of the Weighted Centroid Localization Approach

Before proceeding with the next section, we investigate some implications arising from the WCL approach, which will ease the understanding of our assumptions in the next section. We first remark that Equation (16), and hence Equation (15), is the maximum likelihood solution of the following set of |H| equations

(18) $$\sqrt{p_{ap}}z_{ap}=\sqrt{p_{ap}}z+q_{ap},\;\forall ap\in H$$

for finding *z*, where $q_{ap}$ is a zero-mean Gaussian random variable with identical variance for all ap∈H. Equation (18) in fact implies that, according to the WCL approach, the relationship between the received power $p_{ap}$ and the vertical distance between the MS and the ap-th access point, $d_{z,ap}≜\left|z-z_{ap}\right|$, complies with the following formula:

(19) $$d_{z,ap}=\sqrt{\frac{|q_{ap}{|}^{2}}{p_{ap}}}$$

Now, it is easy to verify that the linear scale Equation (19) will coincide with the noise-free logarithmic scale path-loss model of Equation (4) if

1. Path loss exponent in Equation (4) has the value $n_{ap}=2,\;\forall ap\in H$, which is the typical path-loss exponent of free space.
2. $P_{i,ap}$ in Equation (4) relates to $p_{ap}$ as $P_{i,ap}=10\log p_{ap}$.
3. $P_{T_{ap}}$ in Equation (4) relates to random variable $q_{ap}$ as $P_{T_{ap}}=10\log{\left|q_{ap}\right|}^{2}$. This means that $|q_{ap}{|}^{2}$ represents the transmit power of the ap-th AP that is considered as a nuisance parameter here, whose value is not of interest.
4. The distance $d_{i,ap}$ in Equation (4) represents the distance along the *z*-coordinate.

In other words, WCL model Equation (14) can be regarded as a coarse approximation of path-loss model Equation (4) when the four above assumptions are adopted. In fact, the beauty of the WCL approach is that despite its apparent simplicity and it being a coarse approximation of the channel model (which makes it appealing for use in mobile computing devices) its performance is very promising.

### 3.2. Robust Nonlinear Joint Parameter Estimation and Trilateration

Let us denote the location of MS by (x,y,z). Taking into account the discussion from the previous section, we now start from the assumption that the vertical distance between MS and each AP is proportional to the inverse of the square-root of received power, which is

(20) $$|z-z_{ap}|=\frac{c}{\sqrt{p_{ap}}},\;\;\;ap=1,2,\ldots ,N_{ap}$$

This model is in line with the observation made in the previous section and Equation (19), where $c=|q_{ap}|$ is assumed to be the square root of the identical transmit power of APs.

The task is then to estimate *c* and *z* by minimizing the following function

(21) $$F\left(z,c\right)=\sum_{ap=1}^{N_{ap}}f_{ap}{\left(z,c\right)}^{2}$$

where

(22) $$f_{ap}\left(z,c\right)=(|z-z_{ap}\left|-\frac{c}{\sqrt{p_{ap}}}\right)$$

Employing the Gauss–Newton method, we obtain the following iterative formula for finding the minimizer of Equation (21)

(23) $${\hat{\theta }}_{\left\{k+1\right\}}={\hat{\theta }}_{\left\{k\right\}}-{\left(J_{\left\{k\right\}}^{T}J_{\left\{k\right\}}\right)}^{-1}J_{\left\{k\right\}}^{T}f_{\left\{k\right\}}$$

where subscript {k} denotes the value in *k*-th iteration, $\theta ≜{\left[z,c\right]}^{T}$, and

(24) $$\begin{array}{c} J≜\left[\begin{array}{cc} \frac{\partial f_{1}}{\partial z} & \frac{\partial f_{1}}{\partial c}\\ \frac{\partial f_{2}}{\partial z} & \frac{\partial f_{2}}{\partial c}\\ \vdots & \vdots \\ \frac{\partial f_{N_{ap}}}{\partial z} & \frac{\partial f_{N_{ap}}}{\partial c} \end{array}\right] \end{array}$$

with $\frac{\partial f_{ap}}{\partial z}=\mathrm{sign}\left(z-z_{ap}\right)$, $\frac{\partial f_{ap}}{\partial c}=\frac{-1}{\sqrt{p_{ap}}}$, and

(25) $$\begin{array}{c} f={\left[f_{1},f_{2},\ldots ,f_{N_{ap}}\right]}^{T} \end{array}$$

To robustify this algorithm, we notice that the second term in the right hand side of Equation (23) can be interpreted as the LS solution to the regression equation

(26) f=JΔθ+noise

for finding Δθ. Therefore, to robustify Equation (23), we can simply replace this term with the IRLS solution of Equation (26) to get the following formula for the robust joint estimation of *z* and *c*:

(27) $${\hat{\theta }}_{\left\{k+1\right\}}={\hat{\theta }}_{\left\{k\right\}}-\mathrm{robustfit}\left(J_{\left\{k\right\}},f_{\left\{k\right\}}\right)$$

The method can be easily extended to a trilateration case, where all three coordinates (x,y,z) are assumed to affect the received RSS. Although a more realistic assumption, it deteriorates the floor detection results in practice, because of the introduction of the errors along the nuisance directions *x* and *y* to the problem.

### 3.3. Robust Linear Joint Parameter Estimation and Multilateraion

Let us denote the location of MS by (x,y,z), the coordinates of the *i*-th AP by $\left(x_{i},y_{i},z_{i}\right)$, the distance of MS to the *i*-th AP by $r_{i}$, and the distance between the *i*-th and *j*-th APs by $d_{ij}$. Then, it can be easily verified that the set of the following $N_{ap}-1$ equations are satisfied [4].

(28) $$\begin{array}{c} \left\{\begin{array}{ccc} \left(x-x_{1}\right)\left(x_{2}-x_{1}\right)+\left(y-y_{1}\right)\left(y_{2}-y_{1}\right)+\left(z-z_{1}\right)\left(z_{2}-z_{1}\right) & = & 0.5(r_{1}^{2}-r_{2}^{2}+d_{21}^{2})\\ \left(x-x_{1}\right)\left(x_{3}-x_{1}\right)+\left(y-y_{1}\right)\left(y_{3}-y_{1}\right)+\left(z-z_{1}\right)\left(z_{3}-z_{1}\right) & = & 0.5(r_{1}^{2}-r_{3}^{2}+d_{31}^{2})\\ \vdots \\ \left(x-x_{1}\right)\left(x_{N_{ap}}-x_{1}\right)+\left(y-y_{1}\right)\left(y_{N_{ap}}-y_{1}\right)+\left(z-z_{1}\right)\left(z_{N_{ap}}-z_{1}\right) & = & 0.5(r_{1}^{2}-r_{N_{ap}}^{2}+d_{N_{ap}1}^{2}) \end{array}\right. \end{array}$$

Assuming that $r_{i}=\frac{c}{\sqrt{p_{i}}},i=1,\ldots ,N_{ap}$, this can be rewritten in matrix form as

(29) Gx=d

where

(30) $$\begin{array}{c} G≜\left[\begin{array}{cccc} x_{2}-x_{1} & y_{2}-y_{1} & z_{2}-z_{1} & \frac{1}{2}\left(\frac{1}{p_{2}}-\frac{1}{p_{1}}\right)\\ x_{3}-x_{1} & y_{3}-y_{1} & z_{3}-z_{1} & \frac{1}{2}\left(\frac{1}{p_{3}}-\frac{1}{p_{1}}\right)\\ \vdots & \vdots & \vdots & \vdots \\ x_{N_{ap}}-x_{1} & y_{N_{ap}}-y_{1} & z_{N_{ap}}-z_{1} & \frac{1}{2}\left(\frac{1}{p_{N_{ap}}}-\frac{1}{p_{1}}\right) \end{array}\right] \end{array}$$

(31) $$\begin{array}{c} x={\left[x-x_{1},y-y_{1},z-z_{1},c^{2}\right]}^{T} \end{array}$$

and

(32) $$\begin{array}{c} d=\frac{1}{2}{\left[d_{21}^{2},d_{31}^{2},\ldots ,d_{N1}^{2}\right]}^{T} \end{array}$$

Then, the LS solution for joint location estimation and parameter estimation will be

(33) $$\hat{x}={\left(G^{T}G\right)}^{-1}G^{T}d$$

Now, the robust linear trilateration approach can be obtained by replacing the LS solution by the IRLS solution:

(34) $$\hat{x}=\mathrm{robustfit}\left(G,d\right)$$

The above robust method can be simplified for estimating the vertical position by removing the first two columns of G as well as the first two rows of x and then solving the robust estimation problem in Equation (34).

### 3.4. Robust Deconvolution-Based Path-Loss Estimator

In this section, we show how to robustify the deconvolution-based path-loss parameter estimator [5] by employing the IRLS estimator [18,19]. The deconvolution-based approach consists of two main stages: an offline stage, during which we estimate the AP parameters, and an online stage in which we estimate the MS position. We robustify the algorithm by replacing the ordinary LS approach employed in the offline stage (see [5], Section 3) by the IRLS estimator described earlier.

(35) $${\hat{\Theta }}_{i,ap,\mathrm{robust}}=\mathrm{robustfit}\left(H_{i},P_{ap}\right)$$

where $H_{i}$ is the matrix built from the AP parameters as in Equation (11) [5], and $P_{ap}≜{\left[P_{1,ap},P_{2,ap},\ldots ,P_{N_{f},ap}\right]}^{T}$ is the vector whose *n*-th entry $P_{n,ap}$ is the received power of access point ap at fingerprint *n*.

## 4. Measurement-Based Results

In this section, we study the performance of the proposed algorithms through some real-life numerical examples.

### 4.1. Measurement Set-up

The experimental testbed is set-up in four different buildings in our city: University Building 1 (UBldg1), which is a four-storey building inside the university campus; University Building 2 (UBldg2), which is a three-storey building inside the university campus; a six-storey Mall; and a Shopping center (ShCtr), which is a three-storey building. The RSS data collection in each building is accomplished along several tracks (each including tens to hundreds of measurement points) and the probability of floor detection results are computed by averaging the results over all tracks in each building.

**Figure 2:**
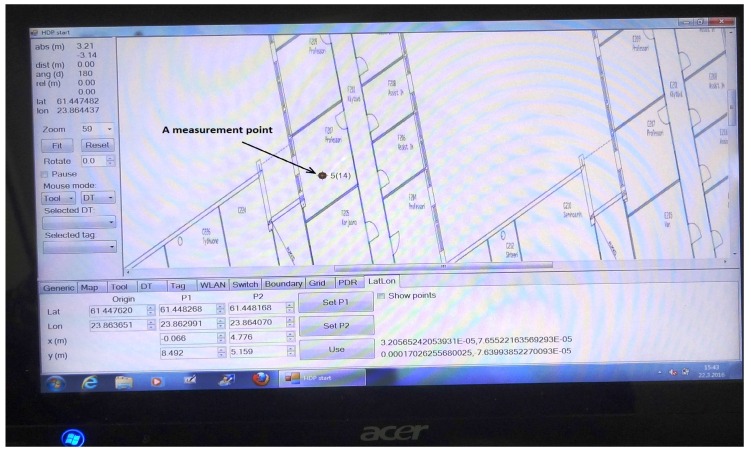
The Acer windows tablet used for collecting data with a sample measurement point shown here. The number outside the parentheses (here, 5) is the fingerprint index, and the number inside (here, 14) shows the number of access points heard in this point.

**Figure 3:**
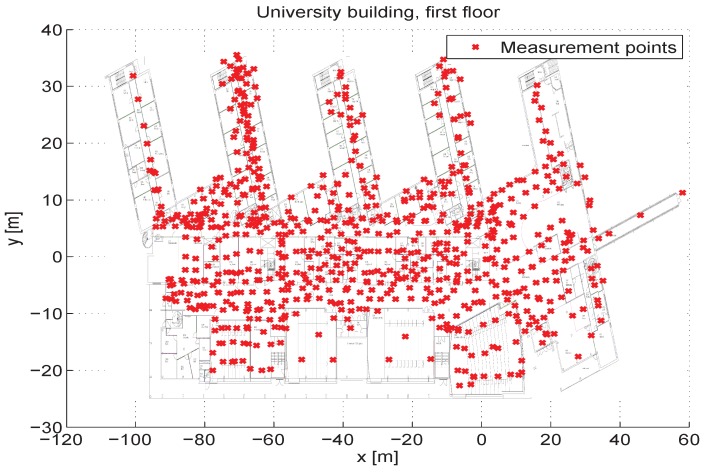
Measurement points in the first floor of UBldg1.

**Figure 4:**
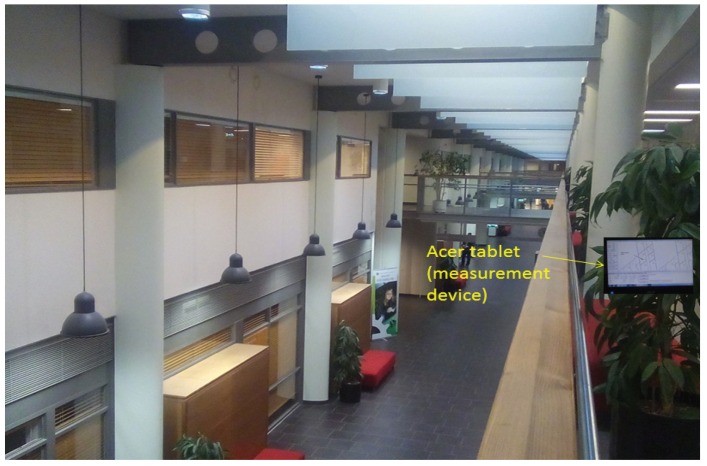
A picture taken from the second floor of Ubldg1.

The device employed for collecting the data is an Acer windows tablet with proprietary software to collect the RSS data, which is pictured in Figure 2. The software records the RSS as well as the coordinates by pressing the location point in the building map. The measurement points in the first floor of Ubldg1 have been shown in Figure 3. To give an idea of the building structure, a picture taken from the second floor of Ubldg1 has been demonstrated in Figure 4.

### 4.2. Numerical Results

The following three examples study the performance of the robust weighted centroid approach, robust deconvolution-based approach, and robust nonlinear trilateration approach, respectively.

*Example 1—Robust weighted centroid localization with real data:* The first example studies the performance of ordinary WCL and robust WCL approaches with four different robust methods: Huber with parameter k=1.345 (Hub1), Huber with parameter k=0.9 (Hub2), Bi-square with parameter k=4.685 (Bsq), and Cauchy with parameter k=2.385 (Cau). The results are shown in Table 3. As can be observed, the robust approaches show improvement over the ordinary WCL approach, regardless of the chosen weighting function.

*Example 2—Robust deconvolution-based approach with real data:* The second example studies the performance of the robust deconvolution-based approach compared to the other deconvolution-based approaches when the estimator employed for parameter vector estimation is least-squares (LS), minimum mean-squared error (MMSE), or weighted least-squares (WLS). The robust function used here is Hub1. The data used here is the same as in the previous example. The results are shown in Table 4.

As can be observed, the robust approach outperforms the other deconvolution-based approaches for all the considered buildings. The only building in which the robust approach is inferior to the others is the Mall, in which all detection methods have rather low detection probabilities.

*Example 3—Robust nonlinear trilateration with real data:* The goal of the third experiment is to study the nonlinear trilateration approach. The data used here is the same as in the previous examples. The deconvolution-based approach has been used for the estimation of AP positions (we use only the *z*-coordinate). We employed the ordinary nonlinear trilateration approach [4], as well as its robust version that we introduced here. The results are shown in Table 5. Again, the results show that robustification has improved the performance. Furthermore, comparison between the second and third columns of the table illustrates how the tuning coefficient affects the performance of the estimator for a given robust weight function. As can be seen, decreasing the value of the tuning coefficient implies more robustification.

*Concluding remarks of the experimental tests:* The results of the three real-life experiments studied in this section demonstrate the ability of the robust methods to improve the performance of localization algorithms used for floor detection in indoor environments. For instance, comparing Hub1 with the ordinary methods in Table 3, Table 4 and Table 5 shows improvement everywhere other than the Mall building for the deconvolution-based method. This is because the Mall building studied in this experiment has a very spacious middle area and therefore can better comply with the Gaussianity assumption in path-loss model, which is the core of the deconvolution-based algorithm.

## 5. Simulation Example

In this section, we study the performance of the proposed robust linear trilateration approach compared to the ordinary linear trilateration [4] through a simulation example. We remark that for the evaluation of this method, we use simulation instead of real data because the linear trilateration is very sensitive to the coordinates of the AP chosen as the reference point, due to the fact that all the distances are determined with respect to this reference point (see, e.g., Equations (28) and (30), where all distances are with respect to AP number 1). In the real data used for the first three experiments, we do not have the exact coordinate of any of the APs to choose it as the reference point. Therefore, the linear trilateration methods are here examined through a simulation example in which the exact location of the APs is assumed known. The number of access points with known locations in the simulation is $N_{ap}=80$, which are located in a four-story building with 20 APs in each floor. The area of each floor is 50 m × 50 m, and the floor height is 4 m. All the APs have the same transmit power and the path-loss exponent is set to $n_{ap}=2,\;\forall ap$. The receiver noise variance varies from 0 to 1. The number of random runs (the random location of MS in the building) is $10^{5}$. The floor detection is performed by first estimating the 3-D coordinates of the MS as in Equation (34) and then rounding the estimated *z*-coordinate to the height of the nearest floor. As can be seen, the robust methods surpass the ordinary LS method. The best performance belongs to Hub2, the Huber robust function with tuning constant k=0.9, which provides more robustness compared to the case k=1.345.

## 6. Conclusions

In this paper, we proposed four robustification methods for floor detection on mobile devices. This was accomplished by exploiting the M-estimators to robustify four existing probabilistic positioning approaches with the goal of applying them to the problem of floor detection from RSS measurements in an indoor environment. The first approach was obtained by robustifying the weighted centroid localization approach by replacing the ordinary sum of squared errors by a more general cost function. The second and third approaches were, respectively, a linear and a nonlinear trilateration approach that first adopt a simplified path-loss model and then estimate the position of MS using robust regression methods. The methods need the information of AP locations as a prerequisite. We finally robustified the deconvolution-based approach [5] via replacing the ordinary least-squares estimator by an iterative reweighted least-squares estimator.

**Figure 5:**
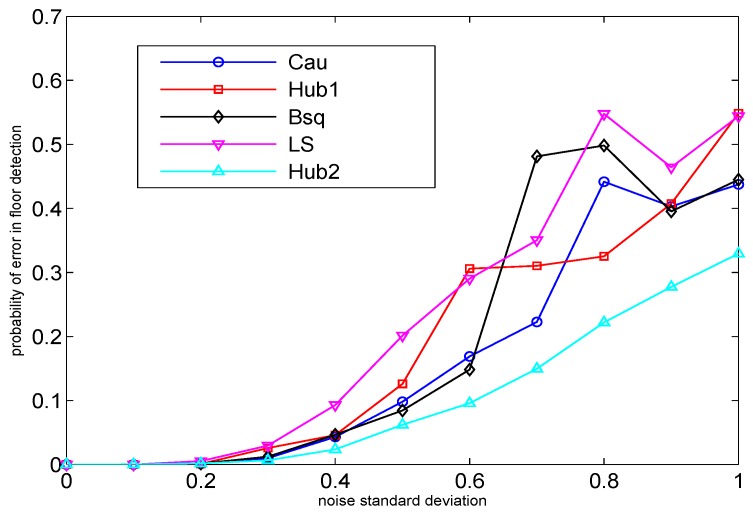
The performance of linear trilateration approaches for different robust weight functions.

The performance of the proposed robust schemes were exemplified both via real-life measurements (Examples 1 to 3 in Section 4.2) and simulations (Figure 5), and all of the proposed robust methods were compared with their non-robust counterparts. In addition, such robust schemes can find their applicability in many LBS applications, such as in emergency applications (e.g., the fast identification of the damaged floors in case of flood or fire), in advertising (e.g., fast finding of the nearby shops at the user’s floor with the desired items), *etc.* In addition, the proposed robustification results can be used jointly with additional sensors, such as barometers, when available on the user’s mobile for increased positioning accuracy.

The main findings of our paper is that, by using robustification, we can achieve better results than without robustification. The proposed mobile computing-based floor detection algorithms, which use the heard RSS information from APs, are indeed feasible with today’s existing WLAN infrastructures.

## Figures and Tables

**Table 1:** Most important positioning-related notations used in this paper. AP: Access point; MS: Mobile station; RSS: Received signal strength.

Quantity	Notation
Number of fingerprints	$N_{FP}$
Number of Access Points	$N_{AP}$
Online 3-D coordinates of MS	c≜(x,y,z)
3-D coordinates of *i*-th access point	$c_{i}≜\left(x_{i},y_{i},z_{i}\right)$
3-D coordinates of *i*-th fingerprint	${\underline{c}}_{i}≜\left({\underline{x}}_{i},{\underline{y}}_{i},{\underline{z}}_{i}\right)$
The RSS of ap-th AP at *n*-th fingerprint	$P_{n,ap}$
The $N_{AP}\times 1$ measurement vector at *n*-th fingerprint	$m_{n}≜{\left[P_{n,1},P_{n,2},\ldots ,P_{n,N_{AP}}\right]}^{T}$
The RSS of ap-th AP received by MS in online phase	$p_{ap}$
The $N_{AP}\times 1$ measurement vector in online phase	$m_{\mathrm{MS}}≜{\left[p_{1},p_{2},\ldots ,p_{N_{AP}}\right]}^{T}$

**Table 2:** Functions ρ(e), ψ(e), and w(e) for ordinary least-squares (LS), and three commonly-used robust functions.

Method	Objective Function ρ(e)	Influence Function ψ(e)	Weight Function w(e)	95% Efficiency Tuning Constant
LS	$e^{2}/2$	*e*	1	none
Huber	$\left|e|\min(|e|,k\right)-\frac{1}{2}{\left(\min(|e|,k)\right)}^{2}$	max[−k,min(k,e)]	min(1,k/|e|)	k=1.345
Bi-square	$\frac{k^{2}}{6}\left(1-{\left({\left(1-{\left(e/k\right)}^{2}\right)}_{+}\right)}^{3}\right)$	$e{\left({\left(1-\frac{e^{2}}{k^{2}}\right)}_{+}\right)}^{2}$	${\left({\left(1-\frac{e^{2}}{k^{2}}\right)}_{+}\right)}^{2}$	k=4.685
Cauchy	$\frac{k^{2}}{2}\log\left(1+{\left(\frac{e}{k}\right)}^{2}\right)$	$\frac{e}{\left(1+{\left(e/k\right)}^{2}\right)}$	$\frac{1}{\left(1+{\left(e/k\right)}^{2}\right)}$	k=2.385

**Table 3:** Comparison of the floor detection probability for different Weighted Centroid Localization (WCL) approaches. Column 2 shows the basic approach, columns 3 to 6 are the robust WCL approach with various robust functions: Hub1 (Huber with tuning coefficient k=1.345), Hub2 (Huber with tuning coefficient k=0.9), Bsq (Bi-square with tuning coefficient k=4.685), and Cau (Cauchy with k=2.385). The highest probability in each row has been shown in Bold. All of the robust methods outperform the ordinary WCL.

Building	WCL	RWCL with Hub1	RWCL with Hub2	RWCL with Bsq	RWCL with Cau
UBldg1	0.852	0.858	**0.866**	0.860	0.860
UBldg2	**0.904**	**0.904**	0.902	0.899	**0.904**
Mall	0.815	**0.839**	0.836	**0.839**	**0.839**
ShCtr	0.827	0.827	0.827	0.827	0.827
Total	0.850	0.857	**0.858**	0.856	**0.858**

**Table 4:** Comparison of the floor detection probability for different deconvolution-based approaches. Columns 2–4 show the basic deconvolution approaches as introduced in [5], and the last column shows the robust deconvolution approach. The robust method outperforms all of the basic deconvolution approaches. MMSE: minimum mean-squared error.

Building	Deconv. LS	Deconv. MMSE	Deconv. WLS	Robust Deconv. with Hub1
UBldg1	0.769	0.771	0.768	**0.818**
UBldg2	0.899	0.907	0.894	**0.925**
Mall	0.448	**0.473**	0.455	0.434
ShCtr	0.649	0.649	0.649	**0.667**
Total	0.665	0.677	0.666	**0.683**

**Table 5:** Comparison of the floor detection probabilities for nonlinear trilateration approach (NTL) and four robust NTL (RNTL) versions of it. All of the robust methods outperform the ordinary nonlinear trilateration.

Building	NTL	RNTL with Hub1	RNTL with Hub2	RNTL with Bsq	RNTL with Cau
UBldg1	0.785	0.790	**0.821**	0.816	0.813
UBldg2	0.685	0.698	**0.725**	0.691	0.695
Mall	0.665	0.697	0.687	**0.700**	**0.700**
ShCtr	**0.796**	**0.796**	0.792	0.792	**0.796**
Total	0.733	0.745	**0.756**	0.750	0.751
